# Inhibition of HDAC increases BDNF expression and promotes neuronal rewiring and functional recovery after brain injury

**DOI:** 10.1038/s41419-020-02897-w

**Published:** 2020-08-18

**Authors:** Naoki Sada, Yuki Fujita, Nanano Mizuta, Masaki Ueno, Takahisa Furukawa, Toshihide Yamashita

**Affiliations:** 1 Department of Molecular Neuroscience, Graduate School of Medicine, Osaka University, 2-2, Yamadaoka, Suita, Osaka 565-0871 Japan; 2 WPI Immunology Frontier Research Center, Osaka University, Suita, Osaka 565-0871 Japan; 3 Department of System Pathology for Neurological Disorders, Brain Research Institute, Niigata University, Niigata, 951-8585 Japan; 4 Laboratory for Molecular and Developmental Biology, Institute for Protein Research, Osaka University, 3-2 Yamadaoka, Suita, Osaka 565-0871 Japan; 5 Graduate School of Frontier Biosciences, Osaka University, 1-3 Yamadaoka, Suita, Osaka 565-0871 Japan; 6 Department of Neuro-Medical Science, Graduate School of Medicine, Osaka University, 2-2, Yamadaoka, Suita, Osaka 565-0871 Japan

**Keywords:** Neurodegeneration, Molecular neuroscience

## Abstract

Brain injury causes serious motor, sensory, and cognitive disabilities. Accumulating evidence has demonstrated that histone deacetylase (HDAC) inhibitors exert neuroprotective effects against various insults to the central nervous system (CNS). In this study, we investigated the effects of the HDAC inhibition on the expression of brain-derived neurotrophic factor (BDNF) and functional recovery after traumatic brain injury (TBI) in mice. Administration of class I HDAC inhibitor increased the number of synaptic boutons in rewiring corticospinal fibers and improved the recovery of motor functions after TBI. Immunohistochemistry results showed that HDAC2 is mainly expressed in the neurons of the mouse spinal cord under normal conditions. After TBI, HDAC2 expression was increased in the spinal cord after 35 days, whereas BDNF expression was decreased after 42 days. Administration of CI-994 increased BDNF expression after TBI. Knockdown of HDAC2 elevated H4K5ac enrichment at the BDNF promoter, which was decreased following TBI. Together, our findings suggest that HDAC inhibition increases expression of neurotrophic factors, and promote neuronal rewiring and functional recovery following TBI.

## Introduction

Traumatic brain injury (TBI) induces severe, long-lasting neurological disabilities, including motor, sensory, and cognitive dysfunctions. Studies support the view that partial functional motor recovery can occur spontaneously after focal cerebral cortex injury^1–6^. Such recovery is correlated with functional organization of remnant neuronal networks^7,8^. It has been shown that reorganization of the corticospinal tract (CST), a major descending motor pathway in mammals which projects from the cortex to the spinal cord, can contribute to post-injury functional motor recovery^9–16^. The CST from the intact side extends axon collaterals into the denervated side of the spinal cord and forms synapses with target neurons, which plays a role in facilitating improved post-injury functional outcomes in mice^11^. During reorganization, remnant CST fibers sprout collaterals, and then, they form synapses with interneurons to construct compensatory neural pathways. The period of spontaneous motor function recovery during which CST fibers sprout can be observed until around 14 days after brain injury in mice. Once synapse formation begins, additional recovery is limited.

We previously reported that brain-derived neurotrophic factor (BDNF) signaling is required for CST fiber rewiring and behavioral recovery post-injury in mice^11^. BDNF expression in the denervated cervical spinal cords was significantly increased 14 days after brain injury; thereafter, the number of fibers recrossing towards the denervated side gradually increased, peaking at day 28^11^. siRNA-mediated BDNF knockdown led to reduce CST axonal branching. However, the molecular mechanisms underlying induction of BDNF expression and synapse formation during CST rewiring remain unclear. Here, we aimed to investigate what mechanism controls the synapse formation, and whether increased synapse formation could contribute to motor function recovery.

Histone deacetylases (HDACs) are enzyme that remove acetyl groups from lysine residues in the amino-terminal tails of histone proteins, leading to chromatin compaction, which is associated with transcriptional and translational repression^17,18^. Structurally, HDACs can be classified into class I (HDAC1, 2, 3, and 8), II (HDAC4, 5, 6, 7, and 9), III (SIRT1–SIRT7), or IV (HDAC11)^19^. Evidence suggests that HDAC expression is altered after central nervous system (CNS) injury, and that HDAC inhibitors can exert neuroprotective effects^20,21^. In particular, HDAC2 has been shown to negatively regulate synaptic plasticity in animal models of neurodegeneration^22^. Increased HDAC2 expression decreased the expression of synaptic plasticity-related genes such as BDNF and synaptophysin. In this study, we assess whether a class 1 HDAC inhibitor (4-acetamido-N-(2-aminophenyl) benzamide [CI-994]) or virus-mediated HDAC2 knockdown enhances synapse formation and motor function of the affected paw after brain injury in mice.

## Materials and methods

### Animals

C57BL/6J mice obtained from Japan SLC, Inc. (Shizuoka, Japan) were bred and maintained at the Institute of Experimental Animal Sciences, Osaka University Graduate School of Medicine. Chx10-CreERT2 transgenic mouse were generated by Dr. Takahisa Furukawa (Osaka University) using bacterial artificial chromosome (BAC) transgenesis. *Cre-ERT2-poly A* signal cassette was inserted into mouse *Chx10* locus. Ai14 female mice (B6.Cg-Gt(ROSA)26Sor^tm14(CAG-tdTomato)Hze^/J)^23^ and R26-CAG-LSL-Sun1-sfGFP-Myc knock-in mice (B6;129-Gt(ROSA)26Sor^tm5(CAG-Sun1/sfGFP)Nat/J^)^24^ were obtained from The Jackson Laboratory for the INTACT method. This study was approved by the institutional committee of Osaka University. All experiments were performed in accordance with the Osaka University Medical School Guide for the Care and Use of Laboratory Animals. Animals were randomized to treatment groups. Experiments, assessments, and analyses were performed in a blinded fashion.

### Surgical procedures for TBI and CI-994 treatment

The hemisphere contralateral to the dominant limb, determined by the single pellet reaching test, was lesioned. Male mice were anesthetized using a mixture of butorphanol (Vetorphale^®^, 0.5 mg/ml, Meiji Seika Pharma, Tokyo, Japan), midazolam, (Dormicum^®^, 0.4 mg/ml, Roche), and medetomidine (Domitor^®^, 0.03 mg/ml, Orion Pharma) via peritoneal injection. Controlled cortical impact (CCI) was then induced as described previously^11^. Briefly, the scalp was retracted, then using a drill and a 23G needle, a 4 mm diameter circular craniotomy was performed on the left side with the center at 0 mm antero-posterior and 2 mm lateral to bregma. Cortical traumatic injury was induced using a pneumatic impact device (Amscien Instruments)^11,13,14^. The impactor tip (diameter, 3 mm) was set at 1 mm, and impact was induced at 4.0–4.5 m/s for 120 ms. Thereafter, the wound was sutured, and the mice were housed in their home cages.

CI-994 (Tokyo Chemical Industry, Japan) was randomly administered as previously described^25^. 10 mg/kg CI-994 was dissolved in the vehicle (dimethyl sulfoxide, DMSO) and administered once daily.

### Immunohistochemistry

Spinal cord sections were prepared from intact and TBI mice. Mice were perfused transcardially with PBS followed by 4% paraformaldehyde in 0.1 M phosphate buffer. Spinal cords were dissected, postfixed in the same fixative, immersed overnight in PBS containing 30% sucrose, and then embedded in Tissue-Tek OCT and frozen at −80 °C until use. Sections were prepared using a cryostat (20 µm thickness) and mounted on Matsunami adhesive-coated slides (Matsunami, Osaka, Japan). Cryostat sections were incubated with blocking solution containing 5% BSA and 0.1% Triton X-100 in PBS for 1 h at room temperature, followed by overnight incubation with primary antibodies (anti-PKCγ, Santa Cruz; anti-GFAP, Sigma-Aldrich; anti-Iba1, Wako, anti-NeuN, Millipore; anti-tdTomato, SICGEN; anti-GFP, Thermo Fischer Scientific; anti-HDAC2, Abcam; anti-HDAC1, Abcam; anti-Chx10, Exalpha biologicals; anti-Olig2, Immuno-Biological Laboratories) at 4 °C. Immunoreactivity was visualized using Alexa Flour 488- or 568-conjugated secondary antibodies (Thermo Fischer Scientific). Coverslips were then placed on the slides with mounting medium (Dako). Nuclei were stained using 4′, 6-diamidino-2-phenylindole (DAPI). Images were captured using a laser scanning confocal microscope (FV-1200, Olympus) or a fluorescence microscope (IX83, Olympus).

### Quantification of lesion volume

Post-injury lesion volume was quantified as previously described^15^. Briefly, coronal brain sections were stained using Cresyl violet (Nissl stain; Sigma-Aldrich). The area of lesioned structures was expressed as a percentage of the volume of corresponding structures in the contralesional hemisphere. Lesion volume was measured using ImageJ (National Institutes of Health, Bethesda, MD, USA); the percentage of the lesion volume was calculated as (contralesional cortex–ipsilesional cortex)/contralesional cortex. To assess CST destruction volume, transverse cervical cord sections were stained using anti-PKCγ antibody. CST area was expressed as a percentage of the PKCγ-immunoreactive area of the uninjured CST (C4–C7). PKCγ staining intensity was measured using ImageJ; the percentage of the lesion volume was calculated as (uninjured CST–injured CST)/uninjured CST.

### Behavioral testing

#### Single pellet reaching test

The single pellet reaching test^26–29^ was used to assess impairment and recovery of forelimb motor function following cortical injury. Briefly, mice were food-restricted to maintain 80–90% of their body weight. The training chamber was a clear acrylic box 13 × 7 × 13.5 cm^3^. A vertical slit (0.5 × 11.5 cm^2^) was located on the front wall of the box. Single chocolate pellets (dustless precision pellets, 20 mg; Bioserv) were placed outside the slit on a platform positioned at a reachable height of 1.5 cm. After two days of food limitation, mice were allowed to reach for multiple pellets presented to them outside the box (days 1–3). On day 4, the dominant limb was defined. Then, individual pellets were placed in front of the slit and animals reached with the dominant limb for a maximum of 30 pellets within 20 min (days 5–14). Animals that consistently using the tongue instead of the forelimb to retrieve pellets were excluded. The success rate was calculated as the percentage of successful reaches out of the total reaching attempts. The percentage success rate for 30 pellets was calculated, and the average percentages of the dominant limb were recorded 7, 14, 21, 28, 35, and 42 days post-injury.

#### Ladder walk test

The ladder walk test was used to assess precise limb placement and stepping while walking along a horizontal ladder with variable rung space^30^. The ladder was designed as previously described^11^. Mice received training three times per session the day before the injury; the percentage of foot-slips for each hindpaw was recorded. The tests started at 2 weeks post-injury and were then performed once a week for four more weeks. The average scores of both hindlimbs were used.

#### Rotarod test

The rotarod is used to assess the motor recovery in rodents after CCI^31,32^. Animals were placed on a rotating rod (diameter 30 mm) that gradually accelerated from 0 to 50 r.p.m. within 5 min. Mice were trained three times a day for 3 days before injury. Total time was recorded until the mouse fell off the rod or gripped and spun around two times. The baseline value (pre) was scored as the mean of three trials 1 day before CCI.

### Cell culture and shRNA

C57BL/6J mouse cortices (E17) were isolated and dissociated with 0.25% trypsin (Invitrogen) and 0.5 mg/mL DNaseI (Sigma-Aldrich) for 15 min at 37 °C. DMEM (Invitrogen) containing 10% FBS was added, and the cells were centrifuged at 1000 rpm for 4 min.

Neurons were transfected with shRNA using Nucleofector^TM^ Solution (Lonza Cologne AG, Cologne, Germany) at room temperature. Thereafter, neurons were plated on poly-l-lysine- and laminin-coated chamber slides and maintained in DMEM/F12 medium containing B27 supplement (Invitrogen) at 37 °C in a 5% CO_2_ atmosphere. shRNA and non-targeting control shRNA sequences were designed as previously described^22,33^. The shRNA sequence was sub-cloned into pSuper-GFP-Neo or pSuper-Neo vectors according to the manufacturer′s protocol (Origene).

Knockdown efficacy was analyzed after 7 days by using real-time PCR. For immunocytochemistry, the cells were cultured for 14 days and fixed with 4% paraformaldehyde for 30 min. Cells were then permeabilized, and non-specific sites were blocked by incubating with PBS containing 0.1% Triton X-100 and 5% BSA. The cells were incubated with anti-GFP (1:2000; Thermo Fischer Scientific) and anti-synapsin (1:1000; Calbiochem) antibodies diluted in a blocking solution overnight at 4 °C. They were then washed in PBS and incubated with fluorescence-conjugated secondary antibodies for 1 h at room temperature. The images were captured using a laser scanning confocal microscope (FV-1200; Olympus). The number of synapsin-positive puncta merged with GFP signals were counted using ImageJ as previously described^34^.

### Semiautomatic analysis of synapses

Neurons were cultured on the myelin-coated plates, and infected with AAV-Syn-tdTomato-T2A-synaptophysin-GFP at DIV 5^35^. The cells were fixed and immunostained with anti-GFP and anti-tdTomato antibodies as described above. The number of synapses was semiautomatically quantified using CellVoyager 8000, a high-throughput cytological discovery system (Yokogawa). Images were collected from five fields per well using a water-immersion lens (x60). Z stacks were done with 0.5-μm steps in z direction. GFP-positive puncta, tdTomato-positive axons, and DAPI-labeled nuclei were semiautomatically traced using consistent fluorescence thresholds for each experimental group. The number of GFP-positive puncta merged with tdTomato-labeled axons were quantified, except the 30-μm area around the DAPI-labeled nuclei of tdTomato-positive neurons. The average of total counts of puncta from five fields are shown in the graph.

### RNA extraction, reverse transcription, and real-time PCR

Total RNA was extracted from cultured neurons, spinal cord, and cortex using TRIzol (Invitrogen), and reverse transcribed using the High-Capacity cDNA Reverse Transcription Kit (Applied Biosystems). Real-time PCR was used to determine mRNA expression (QuantStudio 7 Flex Real-time PCR system; Thermo Fisher Scientific). SYBR Green assay was performed to quantify the expression of indicated targets. Mouse HDACs and BDNF primers were designed as previously described^22,25^. A total of 10 μl was used for SYBR green assays, which contained a 1 × final concentration of Fast SYBR green master mix (Thermo Fisher Scientific). 400 nM gene-specific primers, and 1 μl template. Relative mRNA expression was normalized to the amount of 18 S rRNA in each sample. Cycle threshold values (Ct values) were calculated via the ΔΔCt method to obtain fold differences.

### AAV production

AAVs were produced as previously described^36^. shRNA sequences were designed using previously described sequences^22^. Luciferase-specific shRNA was used as a control. For shRNA-expressing AAV vector production, shRNAs were excised using BamHI and EcoRI and cloned into the pAAV-CMV-tRFP vector. AAV293 cells were transfected with pAAV-shRNA, pHelper, and pack plasmids using the transfection reagent. After a 5-day incubation, the cells were lysed, and the AAV was precipitated with ammonium sulfate solution by centrifugation at 8000 rpm for 70 min. The AAV-containing pellet was washed with PBS and concentrated using Amicon^®^ Ultra-15 10K centrifugal filter devices (Millipore). Two weeks after AAV injection, knockdown efficiency was assessed by real-time PCR. pAAV-phSyn1-tdTomato-T2A-SypEGFP was constructed by modifying AAV- phSyn1(S)-FLEX-tdTomato-T2A-SypEGFP-WPRE^35^.

### AAV injection

For anterograde labeling of synaptic boutons post-injury, pAAV-phSyn1-tdTomato-T2A-SypEGFP was injected into the forelimb area of the intact motor cortex 4 weeks before tissue preparation, as previously described^11^. Briefly, AAV was injected into the forelimb area stereotactically at three sites (coordinates from bregma: 0 mm anterior/1.0 mm lateral; 0.5 mm anterior/1.0 mm lateral, and 0.5 mm anterior/1.5 mm lateral, all at a depth of 0.5 mm) using a glass capillary attached to a microsyringe.

For knockdown experiments, AAV-shRNAs were injected into the spinal cord at cervical level 5–6 after TBI. The virus-infected spinal cord region was confirmed using fluorescence microscopy. Tissues were collected using laser microdissection and subjected to real-time PCR.

### Neuronal isolation

Spinal cord neurons were collected using the INTACT method^24^. Briefly, R26-CAG-LSL-Sun1-sfGFP-Myc knock-in mice were mated with Chx10-CreERT2 mice to enable neuronal nuclear membrane labeling. The cervical spinal cords were collected 42 days post-injury. The tissue was homogenized in ice-cold buffer (0.25 M sucrose, 25 mM KCl, 5 mM MgCl_2_, 20 mM Tricine-KOH) supplemented with 1 mM DTT, 0.15 mM spermine, 0.5 mM spermidine, and EDTA-free protease inhibitor (Roche 11 836 170 001), and lysed in 0.3% IGEPAL-630 buffer. Lysates were mixed with 50% iodixanol density medium (Sigma D1556), layered with a gradient of 30 and 40% iodixanol, and centrifuged at 10,000*g* for 18 min in a swinging bucket centrifuge at 4 °C. After pre-clear, the lysates were immunoprecipitated using monoclonal anti-GFP antibody (Life Technologies G10362) and Dynabeads. The percentage of GFP-expressing nuclei before immunoprecipitation were analyzed on a FACSAria II (BD).

### Laser microdissection

AAV-control or Hdac2 shRNA containing tRFP reporter was injected into the spinal cord at cervical level 5–6 after TBI. Collecting tRFP-labeled tissues was performed as previously described^37^. Briefly, spinal cord tissues were immediately frozen on dry ice 42 days after unilateral injections of the AAV vector. Tissue sections were mounted on glass slides with polyphenylene sulfide (PPS) foil (Leica Microsystems). After toluidine blue staining, the tdTomato-positive area was dissected with an LMD 7000 (Leica Microsystems) and transferred into microcentrifuge tube caps filled with 50 μL of TRIzol reagent (Thermo Fisher Scientific).

### ChIP-qPCR

ChIP was performed using a protocol modified from a previously described method^38^. Spinal cord tissue samples were homogenized in cell lysis buffer containing proteinase (cOmplete™, Roche) and phosphatase inhibitors (1 mM Mb-glycerophosphate, 10 mM NaF, 0.1 mM Na_3_VO_4_), and chromatin was sonicated using a Branson Digital Sonifier with 10 strokes. Sheared chromatin was immunoprecipitated using anti-acetyl H4K5 antibodies (Millipore). Input and ChIP fraction DNA was processed and subjected to quantitative PCR using primers specific to the promoter regions of the indicated genes^22,39^. Each ChIP DNA signal was normalized to the input and represented as percentage of input.

### Statistical analysis

All statistical details are described in figure legends. Statistical analyses were performed using GraphPad Prism 7 (GraphPad Software). Quantitative data are expressed as the mean ± standard error of at least three independent experiments. Differences across all groups were analyzed with ANOVA tests followed by post-hoc tests indicated in “Figure Legends” section. Differences between experimental group pairs were analyzed with Student′s *t* tests. Statistical significance was defined as *P* < 0.05.

## Results

### HDAC inhibition increased functional recovery after brain injury

**Fig. 1:**
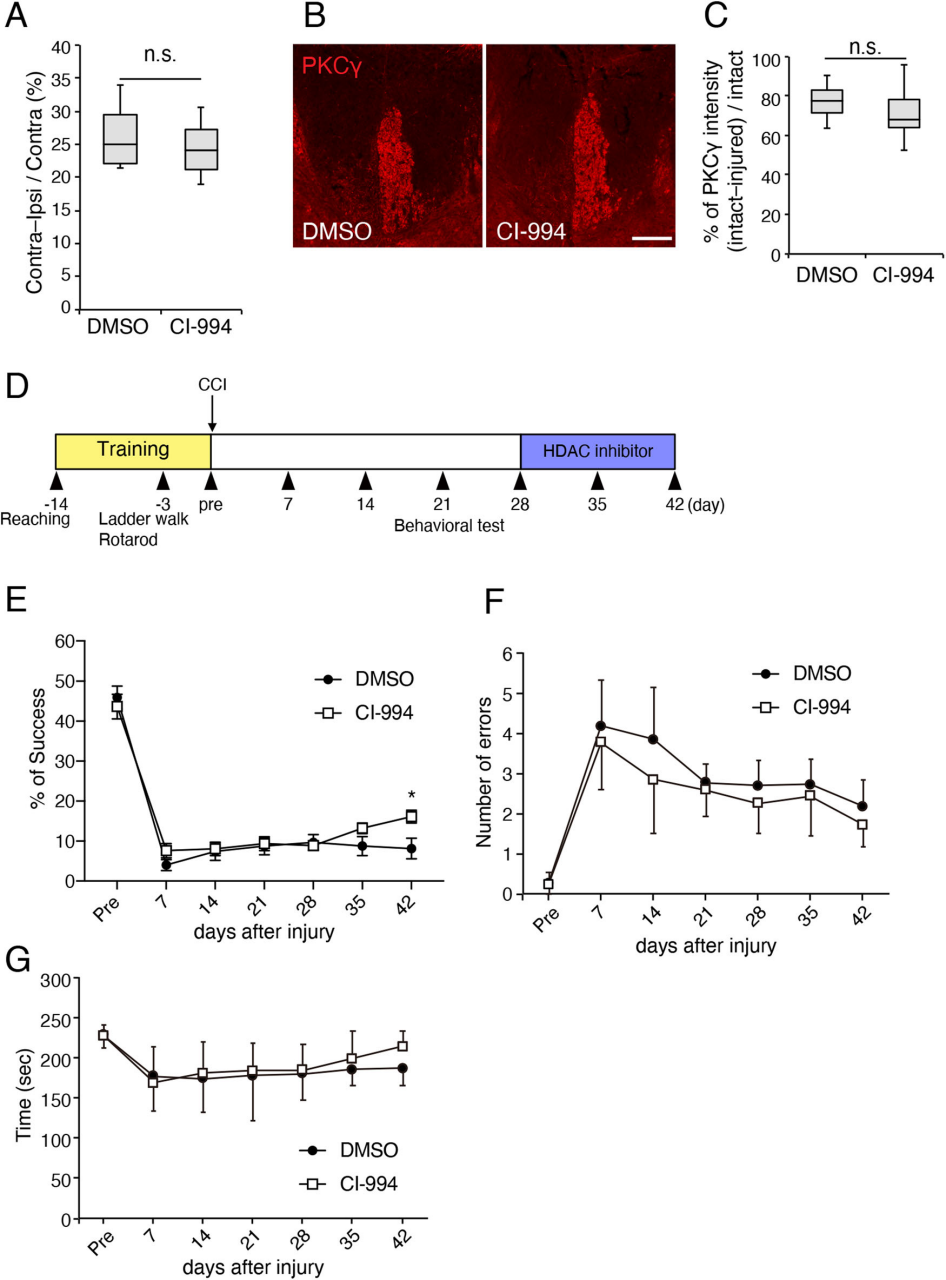
Administration of HDAC2 inhibitor enhances functional recovery after brain injury. **a** Quantitative analysis of the cortical lesion volume in vehicle- (DMSO) or HDAC inhibitor (CI-994)-treated mice. *n* = 4. N.S., not significant; Student′s *t*-test. **b** Representative images of PKCγ immunoreactivity of the dorsal corticospinal tract in the cervical spinal cord. Scale bar: 100 = μm. **c** Quantitative analysis of PKCγ-positive corticospinal tract damage in vehicle-treated and HDAC inhibitor-treated mice. *n* = 14. N.S., not significant; Student′s *t* test. **d** Timeline of CCI and behavior tests. Mice were trained for 14 days for single pellet reaching test, and their behaviors were assessed on indicated days post-injury. (E) Administration of HDAC inhibitor CI-994 from 28 days after injury once a day for 14 days increased success rate for grasping the pellets. *n* = 15. *p < 0.05; Two-way ANOVA with Bonferroni′s post-hoc test. (**f**, **g**) Administration of HDAC inhibitor did not affect the motor behavior examined by the ladder walk (**f**) and rotarod (**g**) tests. *n* = 9. Two-way ANOVA with Bonferroni′s post-hoc test.

To investigate the effect of HDAC inhibition following brain injury, we employed the CCI model^40^. CI-994, a benzamide-based HDAC inhibitor that is relatively selective for class I HDACs^41^, was administrated once daily for 14 days via intraperitoneal injection starting at day 28 post-injury. There were no significant differences in injury volumes between vehicle-treated and HDAC inhibitor-treated mice (Fig. 1a). To determine whether cortical injury degenerated the CST, the cervical spinal cord was stained for protein kinase Cγ (PKCγ), a marker for CST^42,43^. In lesioned mice, PKCγ immunoreactivity was extremely low in the CST originating from the injured cortex. There were no significant differences in CST degeneration between control and HDAC inhibitor-treated mice (Fig. 1b, c). These results confirmed that our CCI model mostly destruct the CST in the injured side and suggest that HDAC inhibition does not affect injury volume.

Using this model, we assessed post-injury motor function of the affected forelimb. Mice were trained to perform a single pellet reaching task for 14 days before injury. They were placed in a box and learnt to use their forelimb to reach and grasp a single food pellet placed beyond a narrow slit and transfer them to their mouth (Fig. 1d). Vehicle-treated mice showed a success rate of ~45% before the injury (Fig. 1e). The success rate decreased by <5% at day 7 post-injury, and then recovered by ~10%. No further increase was observed from day 21 post-injury. In contrast, CI-994 administration increased the success rate of grasping the pellet using their affected forelimb at day42 (Fig. 1e). HDAC inhibition did not promote recovery in other motor tests; the ladder walk and the rotarod test (Fig. 1f, g). These results suggest that HDAC inhibition contributes to functional recovery, especially for grasping tasks, in the late phase following brain injury.

We previously found that CI-994 suppressed immune cell infiltration and neuron loss around the lesion site in early phase following spinal cord injury (SCI)^25^. Lesion volumes did not differ between HDAC inhibitor-treated and vehicle-treated mice following CCI (Fig. 1a). Further, we examined the glial responses in the spinal cord where the remnant CST fibers sprout can be observed following the brain injury. No obvious differences in GFAP-positive or Iba1-positive cell morphology were observed in the cervical spinal cord between the groups at day 42 post-injury (Fig. S1A, B). Nuclei of NeuN-positive neurons were not damaged either (Fig. S1C), and the number was not significantly different between the groups (Fig. S1D). This suggests that HDAC inhibitor administration in the chronic phase post-injury does not significantly affect the lesion volume in the brain and glial response and neuronal survival in the spinal cord.

### HDAC inhibition increased synapse number

**Fig. 2:**
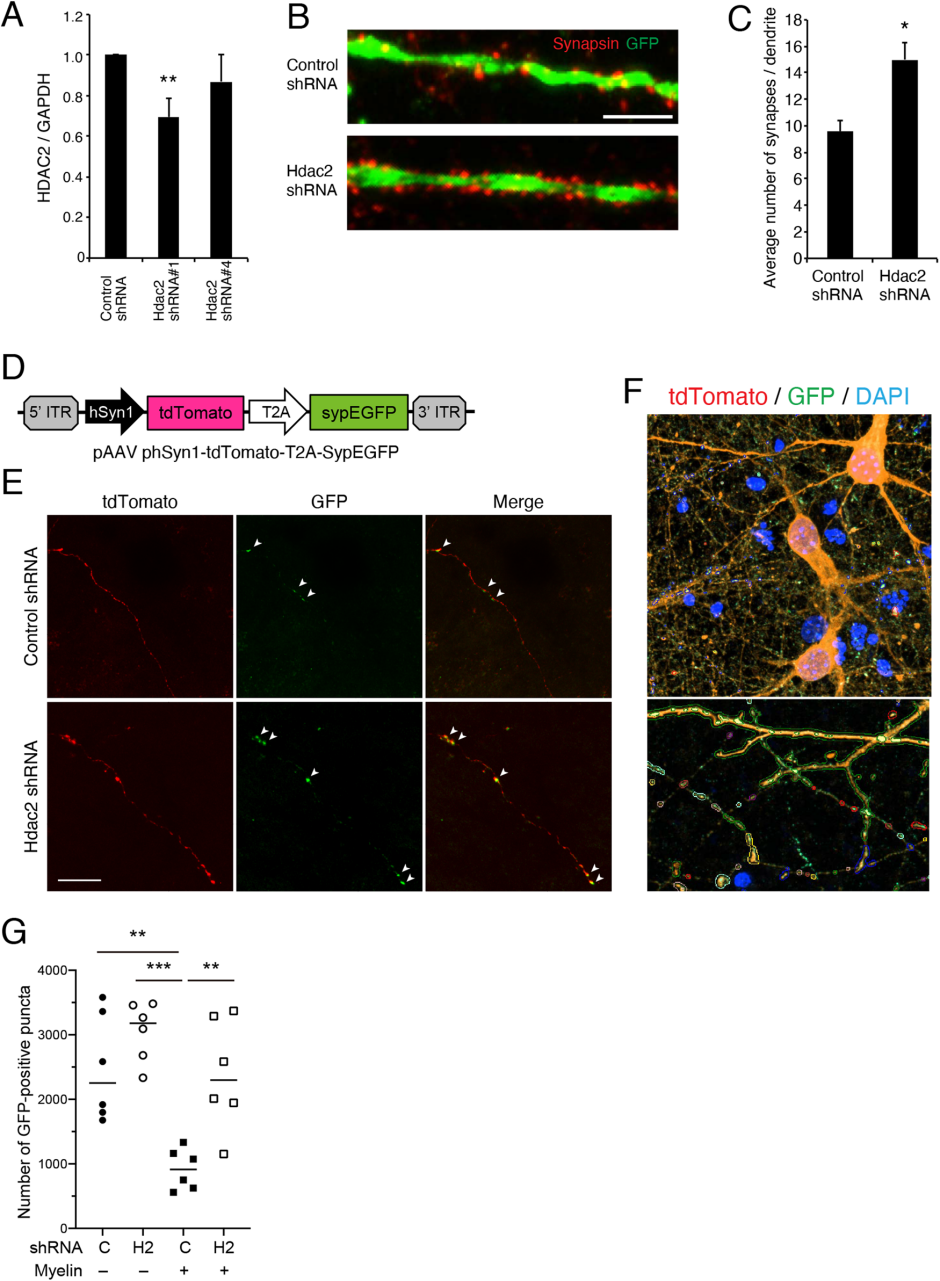
shRNA-mediated knockdown of HDAC2 increases the number of synapses. **a** Knockdown efficiency of HDAC2 shRNA in the cultured cortical neurons. *n* = 4. **p < 0.01; one-way ANOVA with Bonferroni′s multiple comparison test. **b** Representative images of immunocytochemical staining with anti-synapsin and anti-GFP antibodies for the primary cultured neurons transfected with control or HDAC2 shRNA vector containing GFP reporter. Scale bar: 20 μm. **c** Knockdown of HDAC2 increased the number of synapsin-positive puncta on GFP-positive neurons. *n* = 13. *p < 0.05; Welch′s *t*-test. **d** Schematic images of pAAV-phSyn1-tdTomato-T2A-SypEGFP plasmid construction. **e** Representative images showing AAV-labeled axons (red) and synaptic boutons (green) transfected with control or HDAC2 shRNA vector lacking GFP reporter. Arrowheads show synaptophysin-GFP-positive synaptic boutons. Scale bar: 20 μm. **f**, **g** Semi-automated quantitative analysis of the number of GFP-positive synaptic puncta on DIV 14. Representative images of semiautomatic trace were shown in (**f**). HDAC2 shRNA increased the number of synapses cultured on myelin-coated plates (**g**). **c**: control shRNA, H2: HDAC2 shRNA. *n* = 6. **p < 0.01, ***p < 0.005; one-way ANOVA with Bonferroni′s multiple comparison test.

HDAC2 inhibition has been reported to increase synaptic plasticity in neurodegenerative disease models^22^. We therefore assessed the potential effect of HDAC2 on synapse formation using primary cultured cortical neurons. We prepared two HDAC2 shRNA vectors as previously described^22^. HDAC2 shRNA #1 reduced HDAC2 expression in the cortical neurons (Fig. 2a); we therefore used it for the subsequent experiments. shRNA-mediated HDAC2 knockdown increased the number of synapsin-positive punctate signals in Microtubule-associated protein 2 (MAP2)-positive dendrites (Fig. 2b, c), suggesting that HDAC2 inhibition increases synapse number.

We further assessed the effect of HDAC inhibition on synapse formation using semiautomatically quantitative analysis. The neurons were cultured on myelin-coated plate. To visualize the synapses, we infected cultured neurons with adeno-associated viruses (AAVs) encoding tdTomato and synaptophysin-green fluorescent protein (GFP) under the synapsin promoter (pAAV-phSyn1-tdTomato-T2A-SypEGFP) at day in vitro (DIV) 5 (Fig. 2d). We observed GFP-labeled synaptophysin-positive boutons on tdTomato-labeled axons on DIV 14 (Fig. 2e). The number of GFP-positive synaptic boutons was higher in HDAC2 shRNA-transfected neurons compared to that in control shRNA-transfected neurons (Fig. 2f, g). These results suggest that HDAC2 inhibition increases synapse number in vitro.

### HDAC inhibition increased synapse number after brain injury

**Fig. 3:**
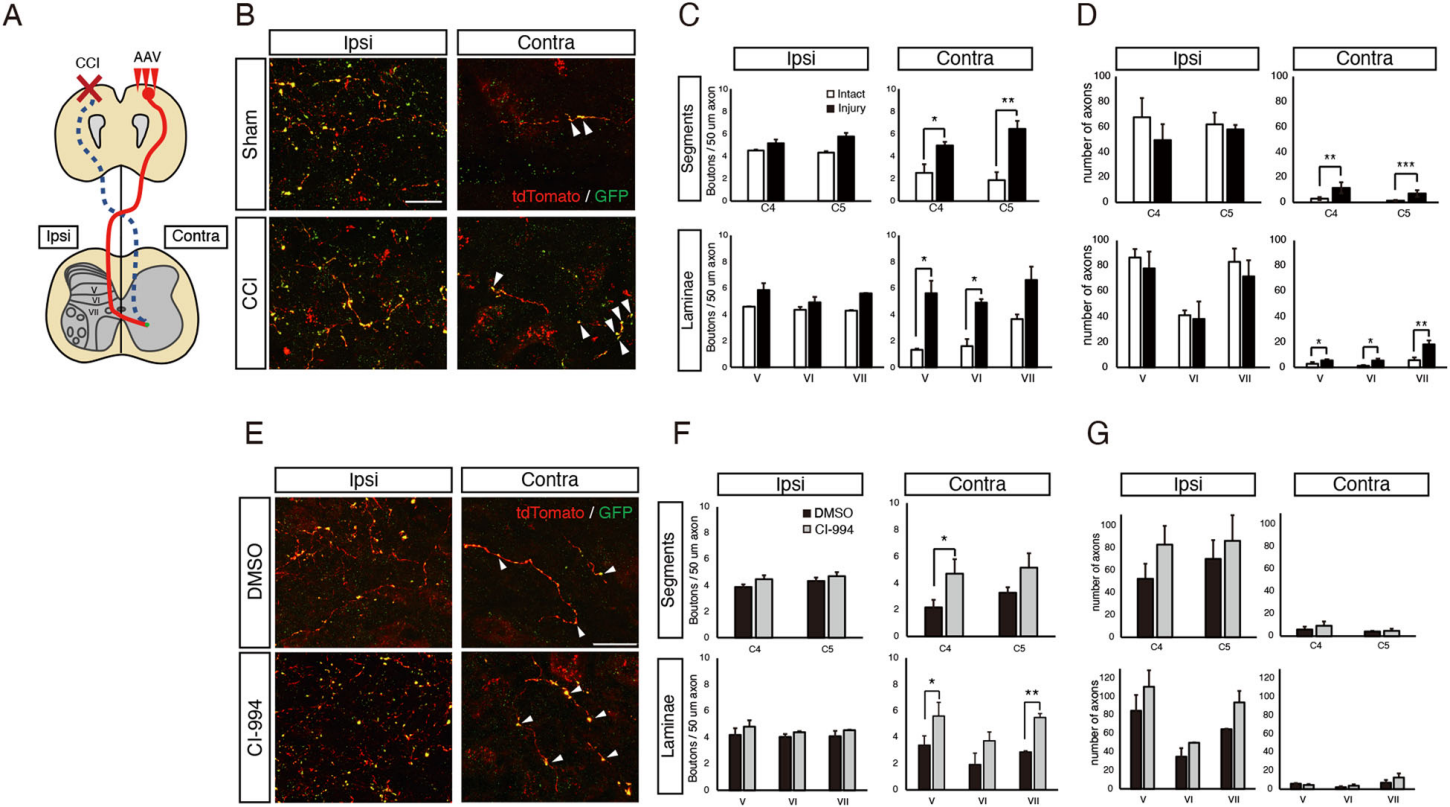
The number of synapses in CST fibers is increased in the denervated side of the cervical spinal cord by HDAC inhibitor-treatment. **a** Schematic illustration of the cortical injury model in this study. Cortical injury to the motor cortex (red) damages the CST (dotted blue lines). AAV-phSyn1-tdTomato-T2A-SypEGFP was injected into the contralesional (uninjured) motor cortex to label the intact CST and synapses. DMSO or CI-994 was injected once a day from 28 days after the injury. The sprouting axons (red line) of the intact CST cross the midline into the denervated side of the spinal cord (Contra) and form synapses, labeled by GFP (green). **b** Representative images showing AAV-labeled CST axons (red) and synaptic boutons (green) in the cervical spinal cord of Sham and CCI mice at day 42 day after brain injury. Arrowheads show synaptophysin-GFP-positive synaptic boutons. Scale bar: 20 μm. **c**, **d** Quantitative analysis of the number of GFP-labeled synapses (**c**) and tdTomato-labeled CST fibers (**d**) in the Ipsi (AAV-injected CST path thorough) or Contra (AAV-uninjected CST path through) side of the cervical cord of the Sham or CCI mice. *n* = 7 (Sham group), 6 (CCI group). *p < 0.05, **p < 0.01, ***p < 0.005; Student′s *t* test. **e** Representative images showing AAV-labeled CST axons (red) and synaptic boutons (green) in the cervical spinal cord of vehicle-treated (DMSO) and HDAC inhibitor-treated (CI-994) mice 42 days after brain injury. Arrowheads show synaptophysin-GFP-positive synaptic boutons. Scale bar: 20 μm. **f**, **g** Quantitative analysis of the number of GFP-labeled synapses (**f**) and tdTomato-labeled CST fibers (**g**) in the Ipsi (AAV-injected CST path thorough) or Contra (AAV-uninjected CST path through) side of the cervical cord of the vehicle-treated (DMSO) and HDAC inhibitor-treated (CI-994) mice. *n* = 5 (DMSO, CI-994). *p < 0.05, **p < 0.01; Student′s *t* test.

Following unilateral brain injury, the remnant CST fibers from the intact side extend axon collaterals into the denervated side of the spinal cord, and they form synapses with interneurons to reconstruct neural circuits^11^. We therefore assessed the effect of HDAC inhibition on synapse formation after brain injury. To visualize synapses post-injury, we injected pAAV-phSyn1-tdTomato-T2A-SypEGFP into the motor cortex of intact hemisphere post-injury^35,44^ (Fig. 3a). We observed GFP-labeled synaptophysin-positive boutons on tdTomato-labeled CST axons in the cervical spinal cord 42 days post-injury (Fig. 3b); the number of GFP-positive puncta and tdTomato-labeled axons in the denervated side of the spinal cord was increased, consistent with the results of a previous study^11^ (Fig. 3c, d). The number of GFP-positive synaptic boutons was increased in the denervated side of the cervical cord of HDAC inhibitor-treated mice compared to that of vehicle-treated mice (Fig. 3e, f). In contrast, the number of tdTomato-labeled axons did not change significantly (Fig. 3g). This suggests that HDAC inhibition increased synapse formation after brain injury.

### Spinal cord HDAC2 expression patterns after brain injury

**Fig. 4:**
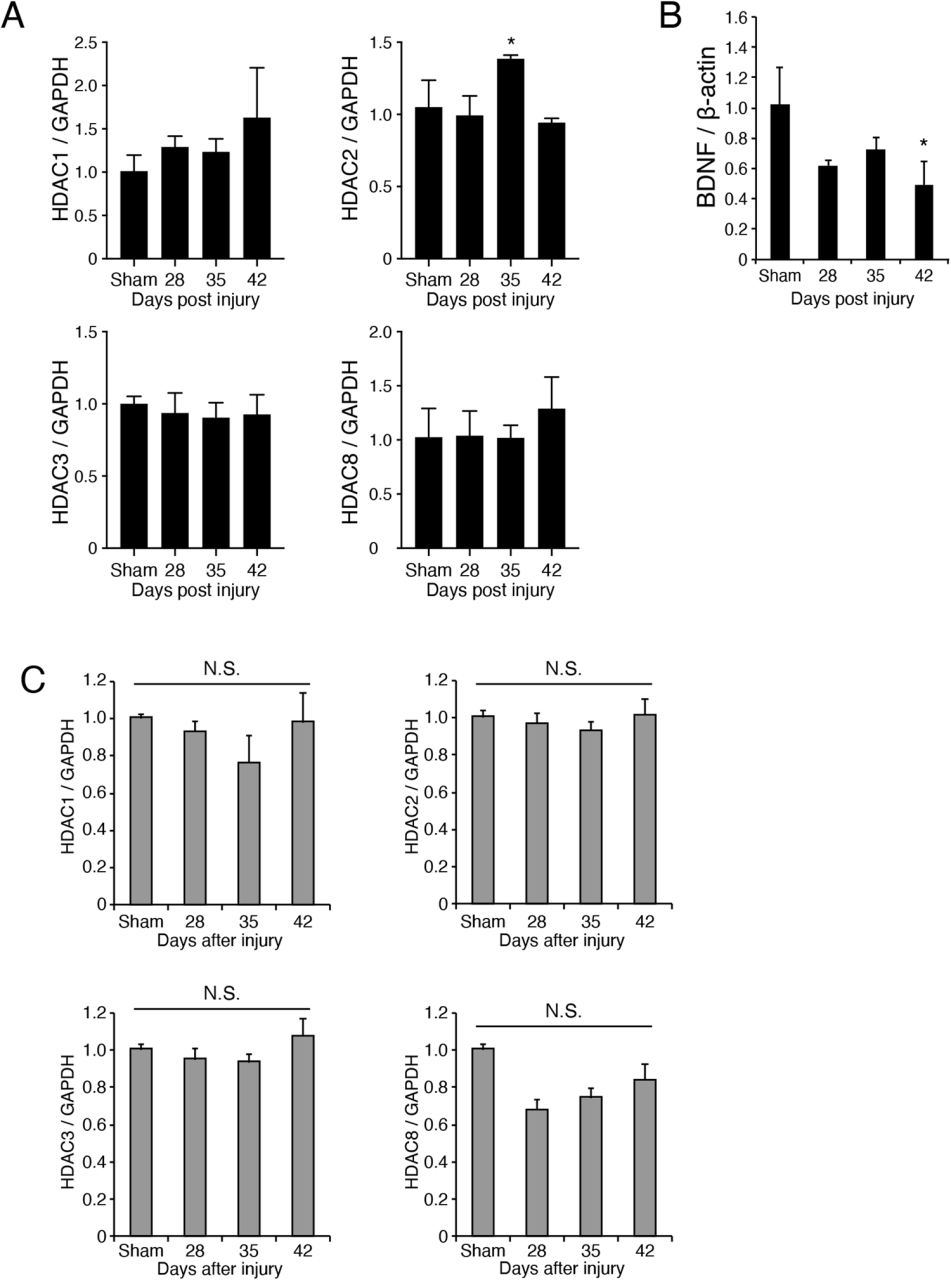
Altered expression of HDAC2 in the denervated side of the cervical spinal cord following injury. **a** Expression changes of class 1 HDACs mRNAs (HDAC1, 2, 3, 8) in the denervated side of the cervical spinal cord following brain injury. *n* = 3. *p < 0.05; one-way ANOVA with Bonferroni′s multiple comparison test. **b** Expression changes of BDNF mRNA in the denervated side of the cervical spinal cord following brain injury. *n* = 5. *p < 0.05; ANOVA with Tukey-Kramer′s multiple comparison test. **c** Expression changes of class 1 HDACs mRNAs in the contralesional motor cortex following brain injury. *n* = 3. *p < 0.05; one-way ANOVA with Bonferroni′s multiple comparison test.

We then quantified the change of class 1 HDACs expressions after brain injury using real-time PCR. HDAC2 expression in the denervated side of the spinal cord was significantly increased 35 days post-injury (Fig. 4a). Conversely, BDNF expression was decreased at day 42 post-injury (Fig. 4b). The time-course dependent change in HDAC2 expression was not detected in the contralesional motor cortex (Fig. 4c). This raises the possibility that HDAC2 upregulation in the denervated spinal cord negatively regulates BDNF expression in the denervated spinal cord.

**Fig. 5:**
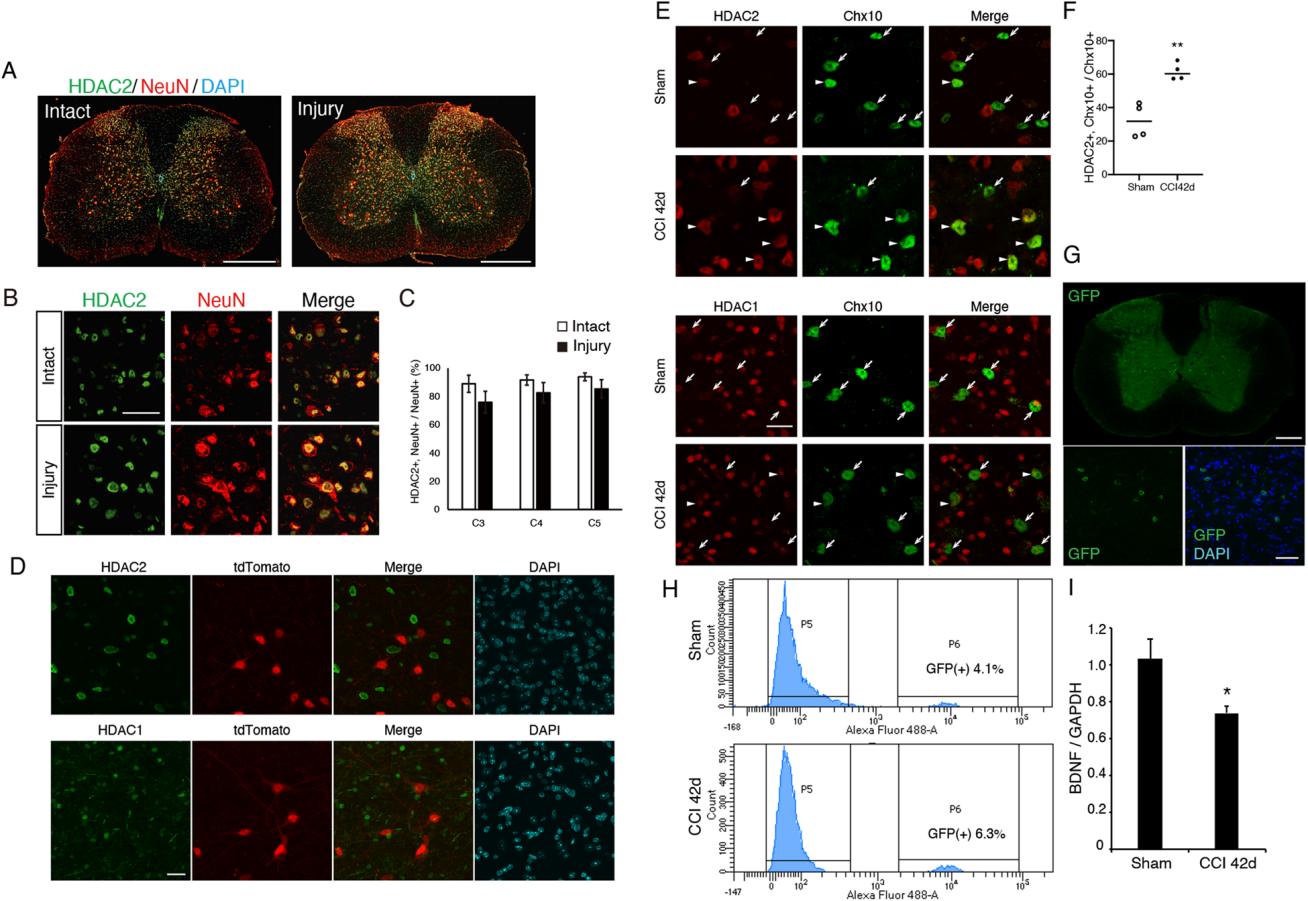
Increased HDAC2 expression in Chx10-positive neurons in the cervical cord. **a**, **b** Representative images of immunohistochemical staining with anti-HDAC2 and anti-NeuN antibodies in transverse sections of the cervical spinal cord (**a**, scale bar: 500 μm) and magnified images of gray matter (**b**, scale bar: 50 μm). **c** No significant difference was detected in the percentage of HDAC2-positive neurons in the denervated side of the cervical cord. *n* = 3. Student′s *t* test. **d** HDAC2 (upper panel) and HDAC1 (lower panel) expression is not detected in Chx10-positive cells in the spinal cord of intact mice. Chx10-CreERT2; Ai14 mice express tdTomato fluorescence in Chx10-positive cells in a tamoxifen-dependent manner. The cervical spinal cord was immunohistochemically stained with anti-HDAC1 or anti-HDAC2 antibodies. Scale bar: 20 μm. **e** The number of HDAC2 and Chx10 double-positive cells appeared to increase 42 days after CCI. Scale bar: 20 μm. **f** The percentage of HDAC2-expressing Chx10-positive neurons in the denervated side of the cervical cord was significantly increased following injury. *n* = 4. Student′s *t* test. **g** Representative images of GFP-labeled Chx10-positive cells by using INTACT method. Scale bar: 200 μm (upper panel), 50 μm (lower panel). **h** The percentage of GFP-positive nuclei collected from the cervical spinal cord (C4–C7) of Chx10-CreERT2; R26-CAG-LSL-Sun1-sfGFP-Myc mice. **i** BDNF mRNA expression in the isolated Chx10-GFP-positive nuclei were significantly decreased in the CCI mice compared with Sham mice. *n* = 3. Student′s *t* test.

We further examined HDAC2 distribution in intact and denervated spinal cord using immunohistochemistry. Most HDAC2-positive cells were localized in NeuN-positive neurons in the gray matter (Fig. 5a). The number of HDAC2-expressing NeuN-positive neurons were quantified; however, no significant difference was detected (Fig. 5b, c). We then examined HDAC2 expression in spinal interneurons where CST axon collaterals potentially project. Various interneuron lineages exist in the spinal cord^45^, of which Chx10-positive neurons expressed HDAC2 (Fig. 5d). To label Chx10-positive cells, we generated Chx10-CreERT2 mice cross-breeding with Ai14 mice^23^. Tamoxifen-dependent Cre recombination causes tdTomato fluorescence expression in Chx10-positive cells. HDAC1 and HDAC2 immunoreactivity was not detected in most tdTomato-labeled Chx10-positive cells in the cervical cord of intact mice (Fig. 5d). In contrast, the number of HDAC2 and Chx10 double-positive cells was significantly increased 42 days post-injury (Fig. 5e, f). We also quantified BDNF expression levels specifically in Chx10-positive neurons. Neuronal nuclei were labeled by using Chx10-CreERT2 mice with the isolation of nuclei tagged in specific cell types (INTACT) method^24^. *Chx10*-GFP-positive nuclei were isolated from the cervical cord of sham and injured mice using fluorescence activated cell sorting, and 4–6% of total nuclei were GFP-positive (Fig. 5g, h). BDNF expression in the *Chx10*-GFP-positive nuclei was significantly decreased in injured mice (Fig. 5i). These results suggest that the increase of HDAC2-expression in Chx10-positive neurons may affect to reduce BDNF expression following brain injury.

**Fig. 6:**
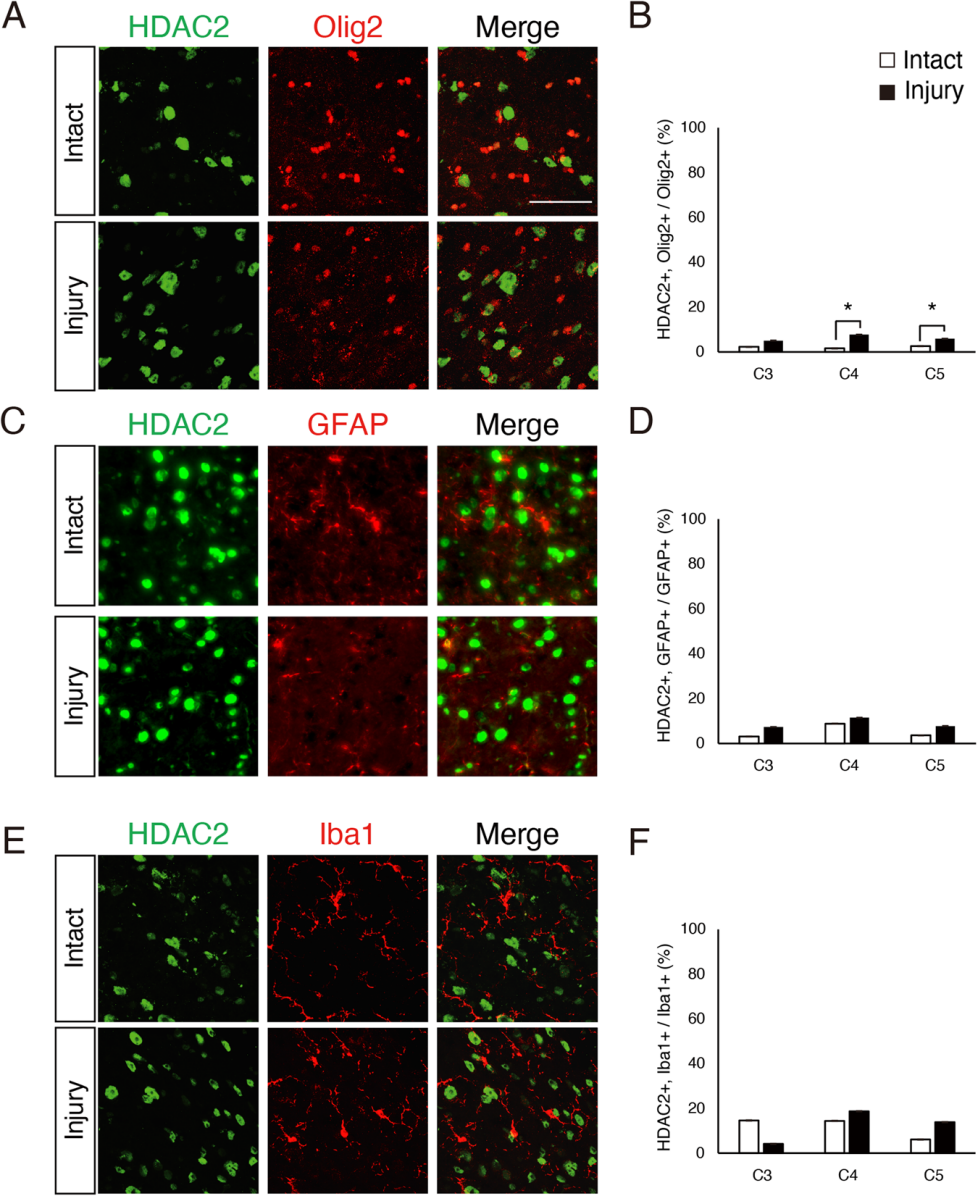
Increased HDAC2 expression in Olig2-positive cells in the cervical cord. **a**, **c**, **e** Representative images of immunohistochemical staining with anti-HDAC2 and anti-Olig2 (**a**), anti-GFAP (**c**), or anti-Iba1 (**e**) antibodies in transverse sections of the cervical spinal cord, scale bar: 50 μm. **b**, **d**, **f** The percentage of HDAC2-expressing cells among the indicated co-labeled cells was quantified in the denervated side of the cervical cord. The percentage of HDAC2-expressing Olig2-positive cells was significantly increased. *n* = 3. Student′s *t*-test.

Furthermore, we examined post-injury HDAC2 expression in the spinal glial cells using immunohistochemistry. The number of HDAC2-expressing Olig2-positive cells was significantly increased post-injury (Fig. 6a, b). However, the number of HDAC2-expressing Iba1-positive microglia or GFAP-positive astrocytes was not significantly different between sham and injured mice (Fig. 6c–f). These results suggest that HDAC2 expression also increased in oligodendrocyte-lineage following brain injury.

### HDAC2 knockdown increased BDNF expression via promoter acetylation after brain injury

**Fig. 7:**
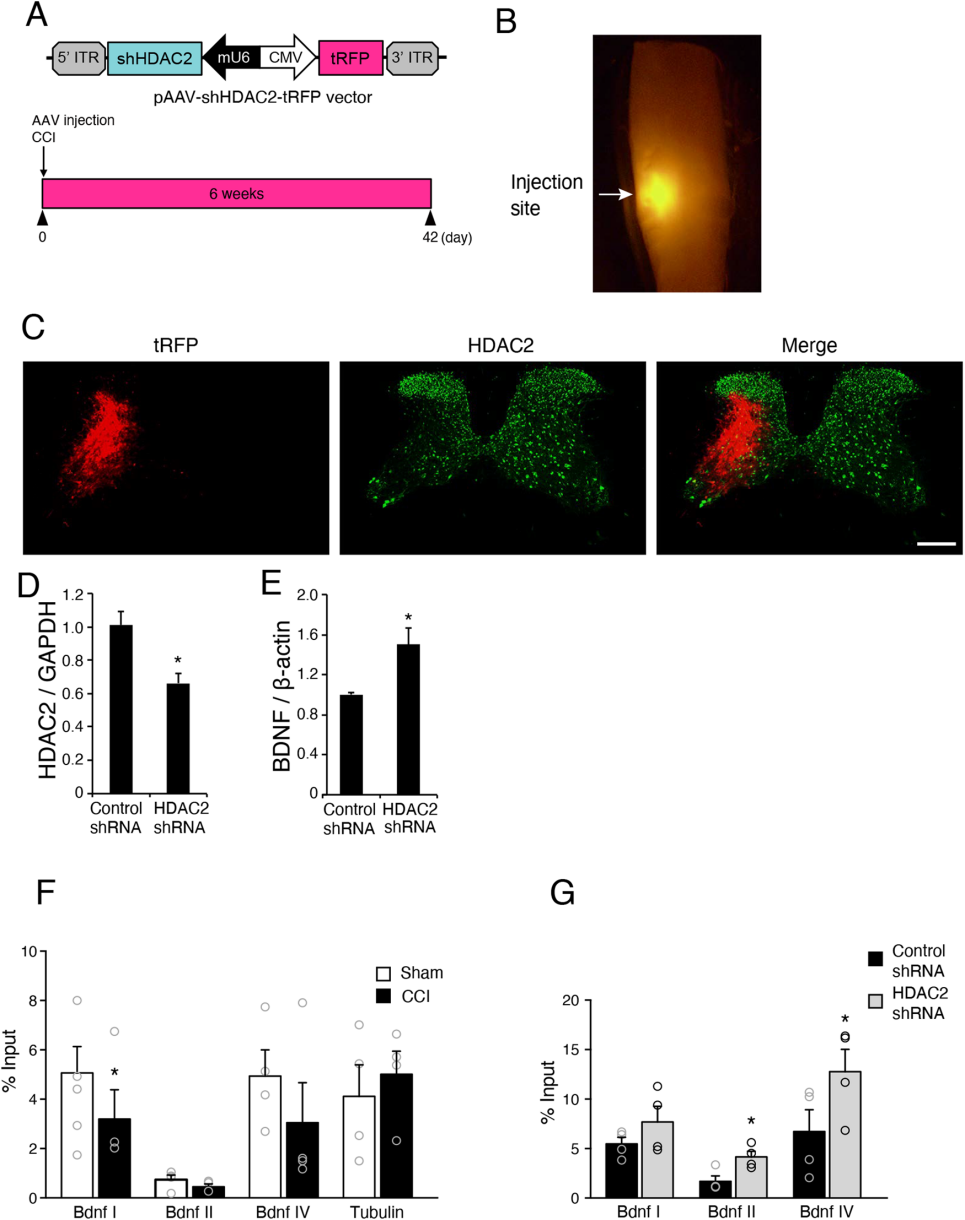
AAV-mediated knockdown of HDAC2 increases BDNF expression following brain injury. **a** Schematic images of AAV-HDAC2 shRNA plasmid construction and timeline of AAV injection in the cervical cord after CCI. **b** Representative images of the cervical spinal cord injected with AAV-shRNA-tRFP. **c** Representative images of transverse sections of the cervical spinal cord injected with AAV-HDAC2 shRNA stained with anti-HDAC2 antibodies. Scale bar: 200 μm. **d** Knockdown efficiency of HDAC2 shRNA in *Chx10*-GFP-positive neurons. Neuronal nuclei were isolated from tRFP-positive regions in the cervical spinal cord following INTACT method, and subjected to RNA extraction and real-time PCR. *n* = 4. *p < 0.05; Student′s *t* test. **e** BDNF expression is increased in *Chx10*-GFP-positive neurons injected with AAV-HDAC2 shRNA compared with control shRNA. *n* = 4. *p < 0.05; Student′s *t* test. **f** Brain injury induces hypoacetylation of H4K5 on BDNF promoter I but not on tubulin promoter in the denervated side of the spinal cord. *n* = 4. *p < 0.05; Student′s *t* test. **g** Injection of AAV-HDAC2 shRNA increases the acetylation of H4K5 on BDNF promoter II and IV following injury. *n* = 4. *p < 0.05; Student′s *t* test.

To examine the association between post-injury HDAC2 and BDNF expression, we used AAV-mediated HDAC2 knockdown in the cervical cord. We injected AAV-expressing shRNA for control or HDAC2 with turbo red fluorescent protein (tRFP) reporter into the level C5–C6 (Fig. 7a, b). The HDAC2 AAV-expressing shRNA-injected side of the spinal cord showed decreased HDAC2 immunoreactivity (Fig. 7c). GFP-positive nuclei labeled with Chx10-CreERT2 mice and the INTACT method were isolated from the spinal cord injected with AAV-shRNA at day 42 after brain injury, and HDAC2 expression was examined using real-time PCR. AAV-HDAC2 shRNA injection significantly decreased HDAC2 expression compared with AAV-control shRNA (Fig. 7d). In these cells, BDNF expression was increased, demonstrating that HDAC2 inhibition enhanced BDNF expression after brain injury (Fig. 7e). These results suggest that HDAC2 negatively regulates BDNF expression during the later stages of the recovery process.

We further examined the mechanism of HDAC2-mediated altered BDNF expression by assessing the acetylation of histone residues in the promoter region of BDNF genes. Acetylation of histone residues, including histone H4 lysine (K)5, has been shown to be important for the effect of HDAC2 on synaptic plasticity^46^. We collected tRFP-labeled area of the spinal cord by laser microdissection. Chromatin immunoprecipitation (ChIP) analysis of tdRFP-labeled samples indicated that H4K5 acetylation of BDNF promoter I was decreased in the denervated side of the spinal cord following brain injury compared to that in intact mice (Fig. 7f). Tubulin promoter H4K5 acetylation was not altered post-injury (Fig. 7f). shRNA-mediated HDAC2 knockdown induced H4K5 hyperacetylation in BDNF promoter II and IV post-injury (Fig. 7g). These results suggest that HDAC2 mediates promoter deacetylation and chromatin compaction of BDNF genes, leading to a decrease in their expression. Lower BDNF expression regulated by HDAC2 may contribute to limited synaptic formation following brain injury.

## Discussion

In this study, we showed HDAC inhibitor administration during the chronic phase after brain injury improved motor function in the affected forelimb. HDAC2 inhibition using AAV-mediated shRNA injection increased H4K5 acetylation of BDNF promoters and expression of BDNF mRNA in the affected side of the cervical cord. Our findings suggest that HDAC inhibition plays crucial roles to increase expression of genes associated with synaptic plasticity and recovery following brain injury.

Behavioral motor outcomes following brain injury have been evaluated using various methods^11,13–15,40^. In the present study, we used the single pellet reaching test to assess the effect of an HDAC inhibitor on the grasping ability of affected forepaw and found that the HDAC inhibitor CI-994 promoted functional recovery (Fig. 1e). HDAC inhibitor also increased the synapse number within the cervical spinal cord (Fig. 3), suggesting that CST rewiring is linked to the recovery of motor function. It has been shown that reorganization within higher levels, such as intracortical^1,2,47^, cortico-rubro-spinal^48,49^, and cortico-reticulo-spinal circuits^50,51^, might also be involved in the recovery process. Although we could not assess the effect of HDAC inhibition on reorganization of these pathways, our present data suggest that enhanced synapse formation at the spinal level could be a candidate of therapeutic targets after brain injury. Behavioral analysis with circuit-specific HDAC2 inhibition could help to reveal the precise effects of HDAC2 on the restorative neural network.

The role of spinal interneurons in controlling mammalian forelimb motor behavior has been examined^52^. More than 20 subtypes of spinal interneurons have been classified during development, and each subtype has specific roles^53–60^. Recently, direct connectivity between corticospinal neurons and spinal pre-motor interneurons, including Chx10-expressing V2a neurons, was identified in mice^45^. Chx10^+^ V2a interneurons are a major ventral excitatory interneuron subtype identified to be involved in reaching behavior^55,61,62^. Genetic ablation of cervical Chx10^+^ interneurons induces deficits in forelimb movement^61^. Furthermore, transplantation of V2a interneurons with neuronal and glial restricted progenitor cells (NPCs) into injured cervical spinal cords exerts greater functional improvement compared to the transplantation of NPCs alone^63^. Spinal cord-derived NPC grafts express motor interneuronal markers including Chx10, and corticospinal axons preferentially regenerate towards graft-derived motor, but not sensory interneurons^64^. These findings provide supporting evidence that Chx10-positive excitatory neurons contribute to post-injury CST rewiring. Here, we found that HDAC2 is expressed in cervical neurons, including Chx10-positive neurons, in the cervical spinal cord. Therefore, it is intriguing to hypothesize that HDAC2 regulates the expression of genes associated with synaptic plasticity in pre-motor interneurons, allowing CST axonal branches to preferentially target these interneurons following CNS injury.

We also demonstrated that the percentage of HDAC2-positive cells increased in oligodendrocyte lineages (Fig. 6a, b). Thus, it will be interesting to assess the role of HDAC2 in BDNF expression in oligodendrocyte lineages. It has been reported that oligodendrocyte-derived-BDNF regulates presynaptic neurotransmitter release via presynaptic tropomyosin receptor kinase B^65^. Furthermore, Olig2 is critical for the development of both motor neurons and oligodendrocyte in the spinal cord^66^. Oligodendrocyte-specific HDAC2 knockdown would be helpful to further assess the post-injury roles of HDAC2.

We previously showed that CI-994 administration also improves functional recovery after SCI^25^. CI-994 was administered 3 h after SCI, and promoted functional motor recovery early on from day 7 post-injury, accompanied by reduced neutrophil infiltration and neuronal loss. This suggests that HDAC inhibition suppresses inflammation and neuronal damage during acute stages. Our present data indicate that CI-994 treatment from day 28 after brain injury increases the synapse number, suggesting that HDAC inhibition promotes CST rewiring during the later stages of recovery. Our studies indicate that CI-994 exerts a protective function against injury, but that the effects differ during the post-injury time-window. Thus, class I HDACs, including HDAC2, regulate diverse processes in various systems, including the nervous and immune systems, during the recovery.

Histone acetylation, which alters the compact chromatin structure to open it up and changes the accessibility of DNA to regulatory proteins, is a fundamental mechanism for regulating gene expression^67,68^. shRNA-mediated HDAC2 knockdown induced BDNF mRNA transcription. Following brain injury, expression of HDAC2, but not other class 1 HDACs, was increased in the affected areas of the cervical spinal cord. These findings suggest that among HDACs, HDAC2 plays a substantial role in the regulation of gene expression related to spontaneous CST reorganization. Importantly, HDAC2 is predominantly localized in NeuN-positive neurons. Furthermore, ChIP-quantitative PCR (qPCR) revealed that HDAC2 inhibition increased BDNF promoter acetylation. Therefore, neuronal HDAC2 might negatively regulate gene expressions related to synaptic plasticity through deacetylation of their promoters, and limit the progression of rewiring.

The role of epigenetic regulation in genes associated with synaptic plasticity, including BDNF, has been shown to be important for learning and memory^69,70^. HDAC inhibitors have been considered as potential therapeutic treatments and shown to be beneficial in reversing cognitive deficits in animal models^18^. TBI also results in substantial cognitive impairments, as well as motor deficits. HDAC inhibition could potentially be a viable therapeutic candidate to treat cognitive and learning dysfunction as well as post-injury motor dysfunction.

Despite the finding that HDAC inhibitor administration increased the synapse number, motor recovery was limited. HDAC inhibitor-treated mice showed a ~20% success rate but not complete recovery (Fig. 1e). Administering HDAC inhibitors immediately after injury may result in both inhibition of inflammation and induced CST rewiring, leading to more effective recovery. Further investigation such as time-dependent and subtype-specific effects of HDAC inhibition would be beneficial to develop therapeutic strategies using HDAC inhibitors to treat brain and SCI.

## Supplementary information

Supplementary Figure Legends

Supplementary Figure

## Data Availability

All data generated or analyzed during this study are included in the manuscript and supporting files.
