# Supplementary material for: Inhibition of HDAC increases BDNF expression and promotes neuronal rewiring and functional recovery after brain injury
Source: Cell Death Dis. 2020 Aug 18;11(8):655. doi: 10.1038/s41419-020-02897-w (PMC7434917; doi:10.1038/s41419-020-02897-w)
Supplement: Supplementary file 1 — Supplementary Figure Legends [file 41419_2020_2897_MOESM1_ESM.docx]

**Supplementary information**

**Inhibition of HDAC increases BDNF expression and promotes neuronal rewiring and functional recovery after brain injury**

Naoki Sada, Yuki Fujita, Nanano Mizuta, Masaki Ueno, Takahisa Furukawa, Toshihide Yamashita

**Supplementary Figure S1. Administration of HDAC inhibitor CI-994 did not affect the number of neurons.**

(A, B) Representative images of immunohistochemical staining of GFAP-positive astrocytes (A), and Iba1-positive microglia/macrophages (B) in the gray matter of the spinal cord following brain injury. Scale bar: 100 μm.

(C) Representative images of immunohistochemical staining of neurons (NeuN) and nuclei (DAPI) in the gray mater following brain injury. Scale bar: 100 μm.

(D) The number of NeuN-positive cells in the spinal cord of vehicle-treated and HDAC inhibitor-treated mice. n = 5. N.S., not significant; Student’s t-test.
