# Supplementary figures and images for: Inhibition of HDAC increases BDNF expression and promotes neuronal rewiring and functional recovery after brain injury
Source: Cell Death Dis. 2020 Aug 18;11(8):655. doi: 10.1038/s41419-020-02897-w (PMC7434917; doi:10.1038/s41419-020-02897-w)

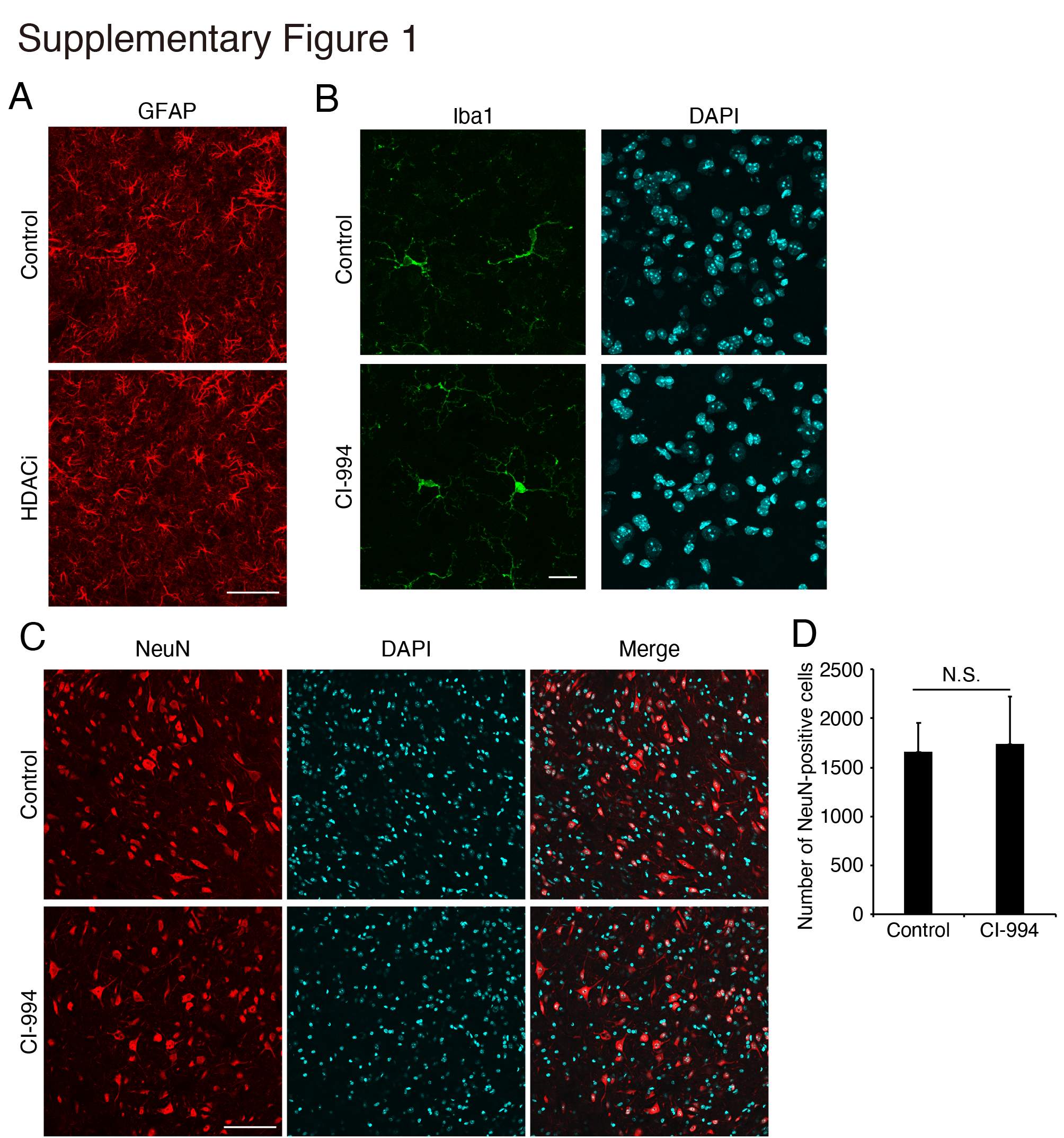

Supplement: Supplementary file 2 — Supplementary Figure [file 41419_2020_2897_MOESM2_ESM.tif]
